# Mitochondrial Fragmentation Is Involved in Methamphetamine-Induced Cell Death in Rat Hippocampal Neural Progenitor Cells

**DOI:** 10.1371/journal.pone.0005546

**Published:** 2009-05-14

**Authors:** Changhai Tian, L. Charles Murrin, Jialin C. Zheng

**Affiliations:** 1 The Laboratory of Neurotoxicology at the Center for Neurovirology & Neurodegenerative Disorders, University of Nebraska Medical Center, Omaha, Nebraska, United States of America; 2 Department of Pharmacology & Experimental Neuroscience, University of Nebraska Medical Center, Omaha, Nebraska, United States of America; 3 Department of Pathology and Microbiology, University of Nebraska Medical Center, Omaha, Nebraska, United States of America; 4 University of Nebraska Medical Center, Omaha, Nebraska, United States of America; National Institutes of Health, United States of America

## Abstract

Methamphetamine (METH) induces neurodegeneration through damage and apoptosis of dopaminergic nerve terminals and striatal cells, presumably via cross-talk between the endoplasmic reticulum and mitochondria-dependent death cascades. However, the effects of METH on neural progenitor cells (NPC), an important reservoir for replacing neurons and glia during development and injury, remain elusive. Using a rat hippocampal NPC (rhNPC) culture, we characterized the METH-induced mitochondrial fragmentation, apoptosis, and its related signaling mechanism through immunocytochemistry, flow cytometry, and Western blotting. We observed that METH induced rhNPC mitochondrial fragmentation, apoptosis, and inhibited cell proliferation. The mitochondrial fission protein dynamin-related protein 1 (Drp1) and reactive oxygen species (ROS), but not calcium (Ca^2+^) influx, were involved in the regulation of METH-induced mitochondrial fragmentation. Furthermore, our results indicated that dysregulation of ROS contributed to the oligomerization and translocation of Drp1, resulting in mitochondrial fragmentation in rhNPC. Taken together, our data demonstrate that METH-mediated ROS generation results in the dysregulation of Drp1, which leads to mitochondrial fragmentation and subsequent apoptosis in rhNPC. This provides a potential mechanism for METH-related neurodegenerative disorders, and also provides insight into therapeutic strategies for the neurodegenerative effects of METH.

## Introduction

Methamphetamine (METH) is abused by over 35 million people worldwide and is an illicit and potent psychostimulant with strong action on the CNS [1], [2]. A serious neuropsychological consequence of METH abuse is cognitive impairment, with working memory deficits remaining long after withdrawal [3]. It is well documented that METH increases glutamate (Glu) levels in the mammalian brain. The high levels of Glu can activate ionotropic receptors, such as N-methyl-D-aspartate (NMDA) and AMPA receptors, resulting in increased intracellular Ca^2+^ levels and formation of reactive nitrogen species (RNS) [4]–[6]. All of these factors contribute to METH-mediated neurotoxicity. In addition, in rodents it has been suggested that METH induces apoptosis of striatal glutamic acid decarboxylase-containing neurons due to the interactions of ER stress and mitochondrial death pathways [7]–[11]. Astroglial activation was also found in METH-induced toxicity [12], [13]. During development and following brain injury, NPC are the source of new neurons and astrocytes in the brain. However, the effects of METH on NPC are not well understood.

The mammalian hippocampus retains its ability to generate neurons throughout life [14]–[16]. Granule neurons are generated from a population of continuously dividing progenitor cells residing in the subgranular zone of the dentate gyrus in the rodent brain [17]. Newborn neurons generated from these progenitor cells migrate into the granule cell layer, differentiate, extend axons and express neuronal marker proteins [18]–[21]. It is also known that the hippocampus is particularly vulnerable to METH. A single challenge of METH suppresses granule cell proliferation in adult gerbils and initiates rewiring of neuronal networks in the prefrontal cortex (PFC), which occurs concurrently with development of severe deficits in PFC-related behaviors. Developmental dysfunction during hippocampal formation is proposed to play a major role in the pathogenesis of neurodegenerative disorders [22]. Defects such as a reduction in hippocampal volume, shape deformations, abnormalities in the granule cell layer, modifications in the mossy fiber pathway, changes in hippocampal cell density and orientation and changes in several cellular markers have been reported [23].

Mitochondria serve as the powerhouse in most eukaryotic cells and play crucial roles in energy metabolism, thermogenesis, maintenance of Ca^2+^ homeostasis and apoptosis [24]. Mitochondria are also dynamic organelles which undergo continuous fission and fusion to form a reticulum structure. Increasing evidence has demonstrated that the changes in mitochondrial morphology depend on the physiological requirements of a cell and are an important determinant of mitochondrial function [25], [26]. In mammalian cells, mitochondrial fission and fusion rely on multiple proteins, such as the dynamin superfamily, which mediate the remodeling of the outer and inner mitochondrial membranes [27]. Among of them, Drp1 [28], [29], Fission 1 (Fis1) [30], [31] and Endophilin B1 (Bif-1/SH3GLB1) [32] control mitochondrial fission. Defects in either mitochondrial fusion or mitochondrial fission may cause severe neurodegenerative diseases [33]–[35].

Recent evidence indicates that mitochondrial dynamics play important roles in the apoptotic process. Many apoptotic stimuli can elicit mitochondrial morphologic changes during the early apoptotic stage, resulting in small, round and more numerous organelles [36]–[38]. Inhibition of mitochondrial fragmentation can not only preserve the mitochondrial architecture but also prevent the release of cytochrome c and subsequent apoptotic steps [39], [40]. How METH regulates the mitochondrial dynamics in rhNPC is not clear.

In this study, we utilized rhNPC as a model and observed that METH induces cell injury of rhNPC, which is accompanied by changes in mitochondrial morphology. Our results further demonstrate that Drp1 not only regulates mitochondrial fragmentation in rhNPC in the same way as described for apoptosis-related fission, but also its oligomerization and translocation from the cytosol to the mitochondria induced by METH contribute to mitochondrial fragmentation. These findings suggest that mitochondrial fragmentation plays an important role in METH-induced rhNPC damage, and they also provide insight into the pathological mechanism of neurodegenerative disorders related to METH abuse.

## Results

### METH inhibits the proliferation of rhNPC

**Figure 1:**
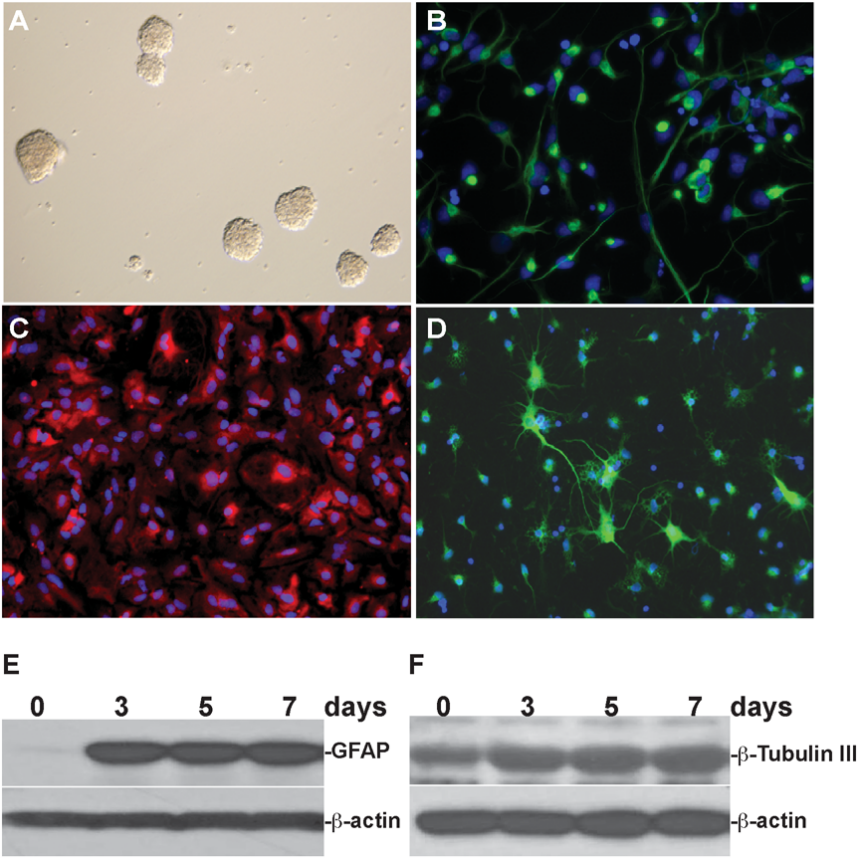
Isolation and differentiation of rhNPC. rhNPC were dissociated from rat hippocampi and cultured with NPBM neural progenitor basal medium containing EGF, bFGF and NSF-1. The neurospheres were formed after culture for 7 days (Fig. 1A). Neurospheres were digested and plated on poly-D-lysine-coated cover slips and cultured with astrocyte differentiation medium or neuron differentiation medium for 7 days and then subjected to immunostaining with nestin (Fig. 1B), GFAP (Fig. 1C) and β-tubulin III (Fig. 1D) antibodies. rhNPC were cultured in six-well plates with differentiation medium for 3, 5 and 7 days, respectively, then subjected to western blotting with GFAP and β-tubulin III antibodies (Fig. 1E, 1F).

We examined the effects of METH on rhNPC derived from the hippocampus of embryonic day 17 (E17) fetus. These rhNPC form neurospheres (Fig. 1A), which are more than 90% nestin positive (a marker for progenitor cells) (Fig. 1B). After changing to specific differentiation medium for astrocytes or neurons and culturing the cells for 7 days, rhNPC appeared to be completely differentiated into astrocytes (Fig. 1C, GFAP as a marker) or neurons (Fig. 1D, β-tubulin as a marker) which was confirmed by immunoblotting (Fig. 1E, 1F).

**Figure 2:**
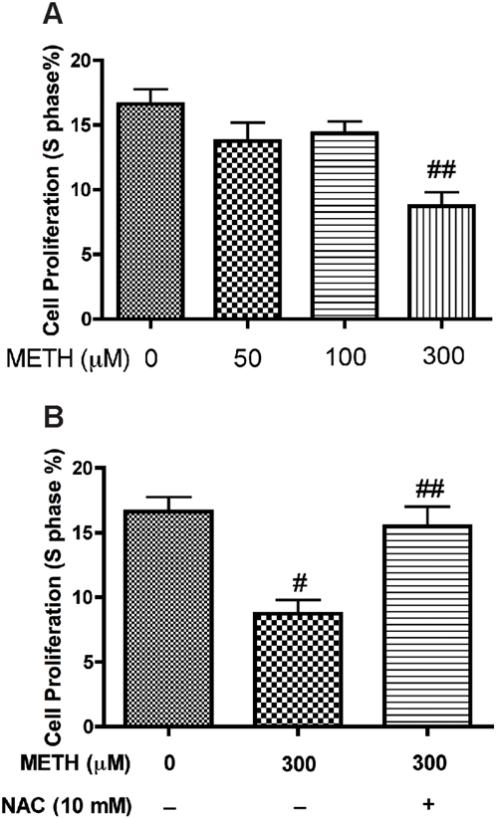
METH inhibits the proliferation of rhNPC. Neurospheres were digested and plated on poly-D-lysine-coated six-well plates, and treated with METH at indicated concentrations for 24 hours followed by cell cycle assays. (A) The population of S-phase cells was analyzed by flow cytometry. The results are expressed as average±SD of triplicate samples from three independent experiments. ## denotes *p*<0.05 in comparison to control. (B) rhNPC were pre-treated with 10 mM NAC for 30 min then treated with 300 µM METH for another 24 hours, and the cell cycle was analyzed by flow cytometry. # denotes *p*<0.05 in comparison to control; ## denotes *p*<0.05 compared with METH alone.

Next, we investigated whether METH affected the neurogenesis of rhNPC. We treated rhNPC with different concentrations of METH for 24 hours, and then performed cell-cycle analysis. The number of proliferating cells decreased in a dose-dependent manner after METH treatment (∼60% decrease, Fig. 2A; p<0.05). In addition, pre-treatment of rhNPC with N-acetyl-cysteine (NAC), a non-specific reductant, reversed this effect (Fig. 2B). These results suggest that METH inhibits the proliferation of rhNPC in an oxidative stress-dependent manner.

### The inhibition of rhNPC by METH is associated with cell apoptosis and accompanied by mitochondrial fragmentation

**Figure 3:**
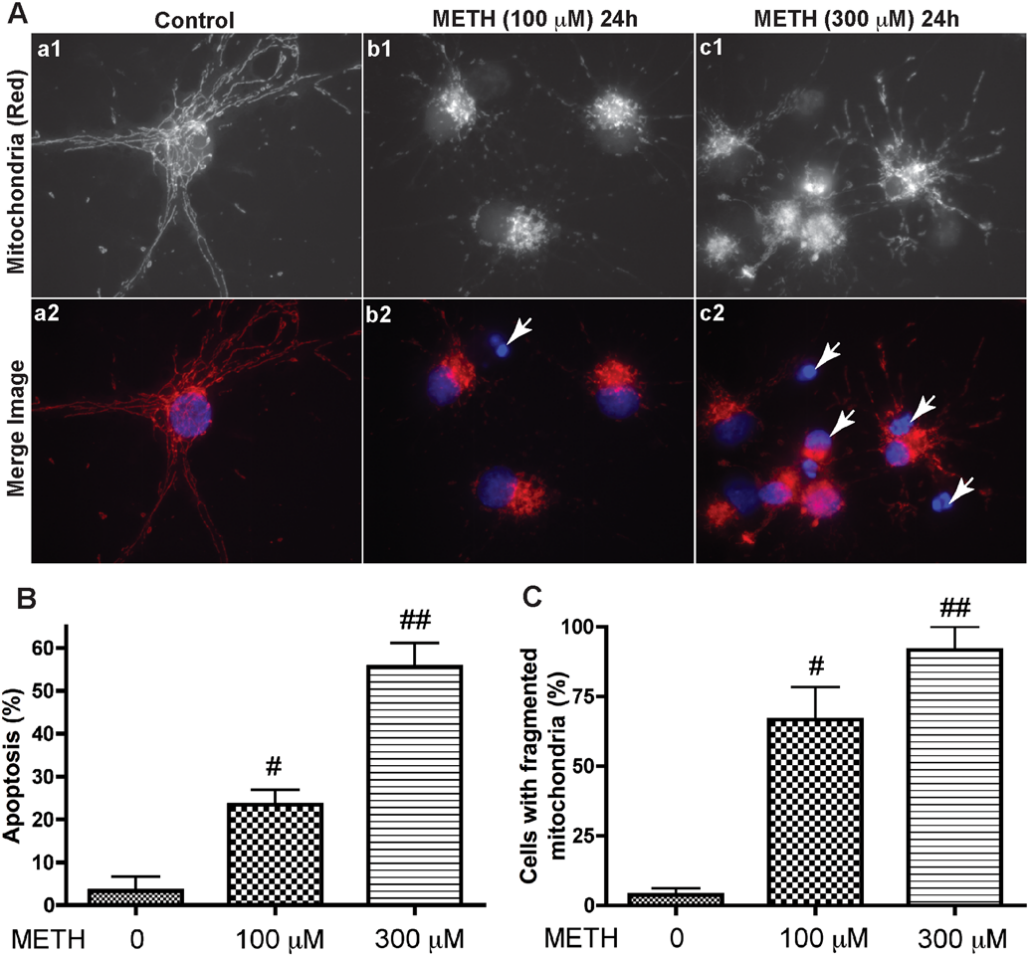
METH-induced apoptosis of rhNPC is accompanied by mitochondrial fragmentation. Neurospheres were digested and plated on poly-D-lysine-coated cover slips. After 24 hours rhNPC were treated with METH at indicated concentrations for another 24 hours. (A) NPC were stained with MitoTracker®Red CMXRos dye (red) and fixed with 3.7% formaldehyde. After permeabilization with 0.2% Triton X-100 in PBS at 4°C for 10 min, cells were subjected to immunostaining with monoclonal nestin antibody and nuclei were stained with DAPI (blue), then visualized by Zeiss Axiovert microscope. (B) Apoptotic nuclei, showing highly condensed and fragmented chromatin, as indicated with arrows in A-b2 and A-c2. The results are expressed as average±SD of triplicate samples. # denotes *p*<0.05 in comparison to control; ## denotes *p*<0.001 compared with control. (C) Cells were analyzed for mitochondrial morphology by fluorescence microscope using MitoTracker®Red. Typical mitochondrial phenotypes (A-a1) and fragmented mitochondria (A-b1 and A-c1) were quantified by counting cell number with or without fragmented mitochondria. # denotes *p*<0.01 in comparison to control; ## denotes *p*<0.001 compared with control.

To investigate the mechanism by which METH inhibits rhNPC proliferation, we examined rhNPC apoptosis induced by different concentrations of METH by nuclear staining. The number of condensed nuclei demonstrating apoptosis was significantly increased with the increasing METH concentrations (Fig. 3A, b2 and c2, indicated by arrow; Fig. 3B). Increasing evidence indicates that mitochondrial dynamics play important roles in the apoptotic process. Many apoptotic stimuli elicit mitochondrial morphologic changes during early apoptosis, resulting in small, round and more numerous organelles [36]–[38]. To examine if the mitochondrial morphological changes are also found in METH-induced rhNPC apoptosis, we stained mitochondria with MitoTracker®Red in addition to DAPI staining. The MitoTracker staining suggests that the mitochondrial network is severely damaged after METH treatment (Fig. 3A, b1 and c1) and the number of cells with fragmented mitochondria increases with increasing concentrations of METH (Fig. 3C).

**Figure 4:**
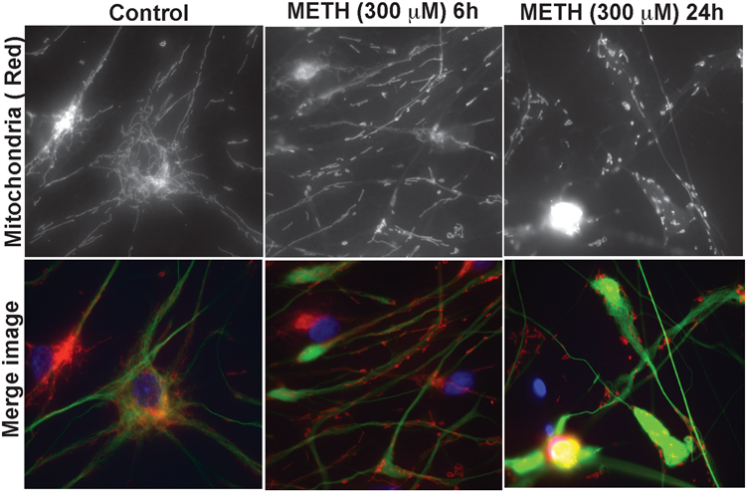
METH induces mitochondrial fragmentation in rhNPC in a time-dependent manner. Neurospheres were digested and plated on poly-D-lysine-coated cover slips. After 24 hours NPC were treated with 300 µM METH for the indicated times and then subjected to mitochondrial staining with MitoTracker®Red (upper panel) and immunostaining with nestin monoclonal antibody (green) and nuclear staining with DAPI (blue) and visualized by Zeiss Axiovert microscope.

To investigate whether the METH-induced mitochondrial fragmentation is related to the duration of METH exposure, we treated rhNPC with 300 µM METH for different time periods and then stained mitochondria with MitoTracker®Red and rhNPC with nestin monoclonal antibody as indicated in Fig. 4. The results demonstrate that prolonged treatment with METH increases the extent of mitochondrial fragmentation (Fig. 4). Taken together, these results suggest that mitochondrial fragmentation is involved in METH-induced NPC apoptosis in a dose- and time-dependent manner.

### Drp1 is involved in the regulation of METH-mediated mitochondrial fragmentation in rhNPC

**Figure 5:**
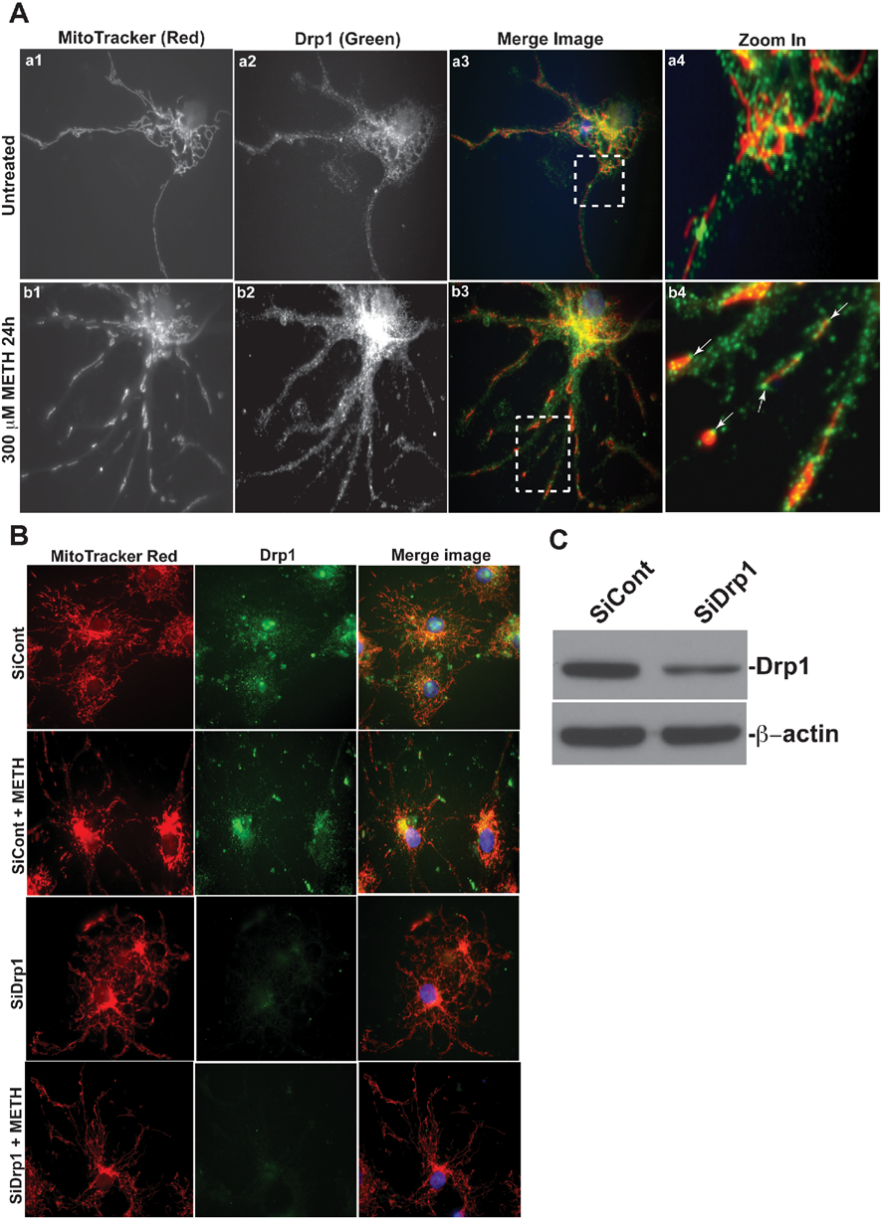
Drp1 is involved in the regulation of METH-induced mitochondrial fragmentation in rhNPC. (A) Neurospheres were digested and plated on poly-D-lysine-coated cover slips, and treated with 300 µM METH for 24 hours then subjected to mitochondrial staining with MitoTracker®Red and immunostained with Drp1 monoclonal antibody (green). (B) rhNPC were transfected with synthesized control-siRNA and Drp1-siRNA on poly-D-lysine-coated cover slips, and 48 hours post-transfection, cells were treated with 300 µM METH for another 24 hours and then subjected to mitochondrial staining and immunostaining with Drp1 monoclonal antibody. (C) rhNPC were transfected with synthesized control-siRNA and Drp1-siRNA on poly-D-lysine-coated cover slips, and 48 hours post-transfection cells were subjected to western blot analysis with Drp1 monoclonal antibody, and β-actin was used as a loading control.

Mitochondrial fission and fusion rely on the function of multiple proteins that mediate the remodeling of the outer and inner membranes [27]. In mammalian cells, at least two proteins, Drp1 and Fis1, are required for mitochondrial fission [41], [42]. Recent evidence indicates that Drp1 is an essential mediator of apoptosis-related mitochondrial fission, and during apoptosis translocates from the cytoplasm to mitochondria, where it forms clusters at prospective sites of mitochondrial fission [39], [43], [44]. Elimination of Drp1 activity during apoptosis results in a block of mitochondrial fission, which leads to long interconnected organelles and a delay in cell death. To evaluate the contribution of Drp1 in regulating mitochondrial morphology during METH treatment, we examined the expression and localization of Drp1 in rhNPC. The results show that under normal conditions, Drp1 has a punctate distribution in the cytoplasm around mitochondrial networks (Fig. 5A, upper panel); however, after treatment with 300 µM METH for 24 hours, the distribution of Drp1 in the cytoplasm was mainly localized at both ends of the mitochondrial fragments (Fig. 5A lower panel: indicated by arrows in b4). These data suggest that Drp1 is involved in METH-induced mitochondrial fragmentation. To further confirm this possibility, we down-regulated Drp1 expression in rhNPC by siRNA duplexes targeted against rat Drp1 mRNA, and then examined the expression of Drp1 by western blotting (Fig. 5C) and mitochondrial morphology after METH treatments (Fig. 5B). The results demonstrate that mitochondrial structure in the Drp1 siRNA-transfected cells still remains connected with mitochondrial networks despite METH treatment (Fig. 5B-c1 and d1); however, mitochondria undergo fragmentation in siRNA control-transfected cells after METH treatment (Fig. 5B-b1). These results further support the notion that Drp1 plays an important role in METH-mediated mitochondrial fragmentation.

### Oxidative stress, but not Ca^2+^ influx, is involved in METH-mediated mitochondrial fragmentation in rhNPC

**Figure 6:**
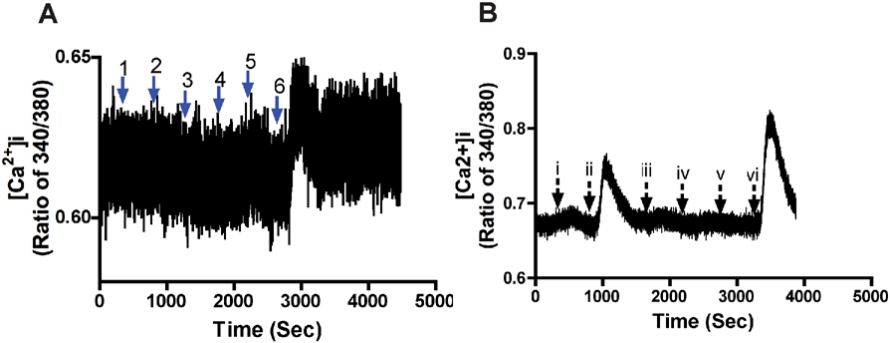
METH has no effect on Ca^2+^ influx or glutamate-mediated Ca^2+^ influx in rhNPC. rhNPC cultured on poly-D-lysine-coated cover slips (25 mm), were loaded with Fura-2 and monitored for calcium influx by micro-fluorescent imaging. (A) Different concentrations of METH were tested and calcium influx was measured. METH concentrations were 1: 50 µM; 2: 100 µM; 3: 500 µM; 4: 1 mM; 5: 5 mM; 6:100 µM glutamate as a positive control. (B) METH has no effect on glutamate-mediated AMPA receptor responses; a: glutamate (100 µM); b: glutamate (100 µM)+cyclothiazide (10 µM); c: glutamate (100 µM)+cyclothiazide (10 µM)+CNQX (10 µM); d: glutamate (100 µM)+500 µM METH+cyclothiazide (10 µM).

It is well documented that METH increases Glu levels in the mammalian brain, which can activate ionotropic receptors, such as NMDA and AMPA receptors, resulting in increased intracellular Ca^2+^ levels and ROS formation [4]–[6]. All of those factors contribute to METH-mediated neurotoxicity. To investigate the effects of METH on intracellular Ca^2+^ levels and ROS, we first examined Ca^2+^ influx in rhNPC by loading them with Fura-2 and monitoring Ca^2+^ influx by live-fluorescent imaging. Using Ca^2+^ imaging on groups of 20 rhNPC, we found that METH had no effects on Ca^2+^ influx, even after treatment with 5 mM METH (Fig. 6A). Moreover, we observed that 100 µM Glu stimulation caused Ca^2+^ influx, and the observed Ca^2+^ influx was greatly increased using the AMPA receptor-specific potentiator, cyclothiazide, but it was almost completely blocked by AMPA/kainate receptor antagonist, CNQX [45]. However, METH had no effects on Glu-mediated Ca^2+^ influx through AMPA receptors (Fig. 6B).

**Figure 7:**
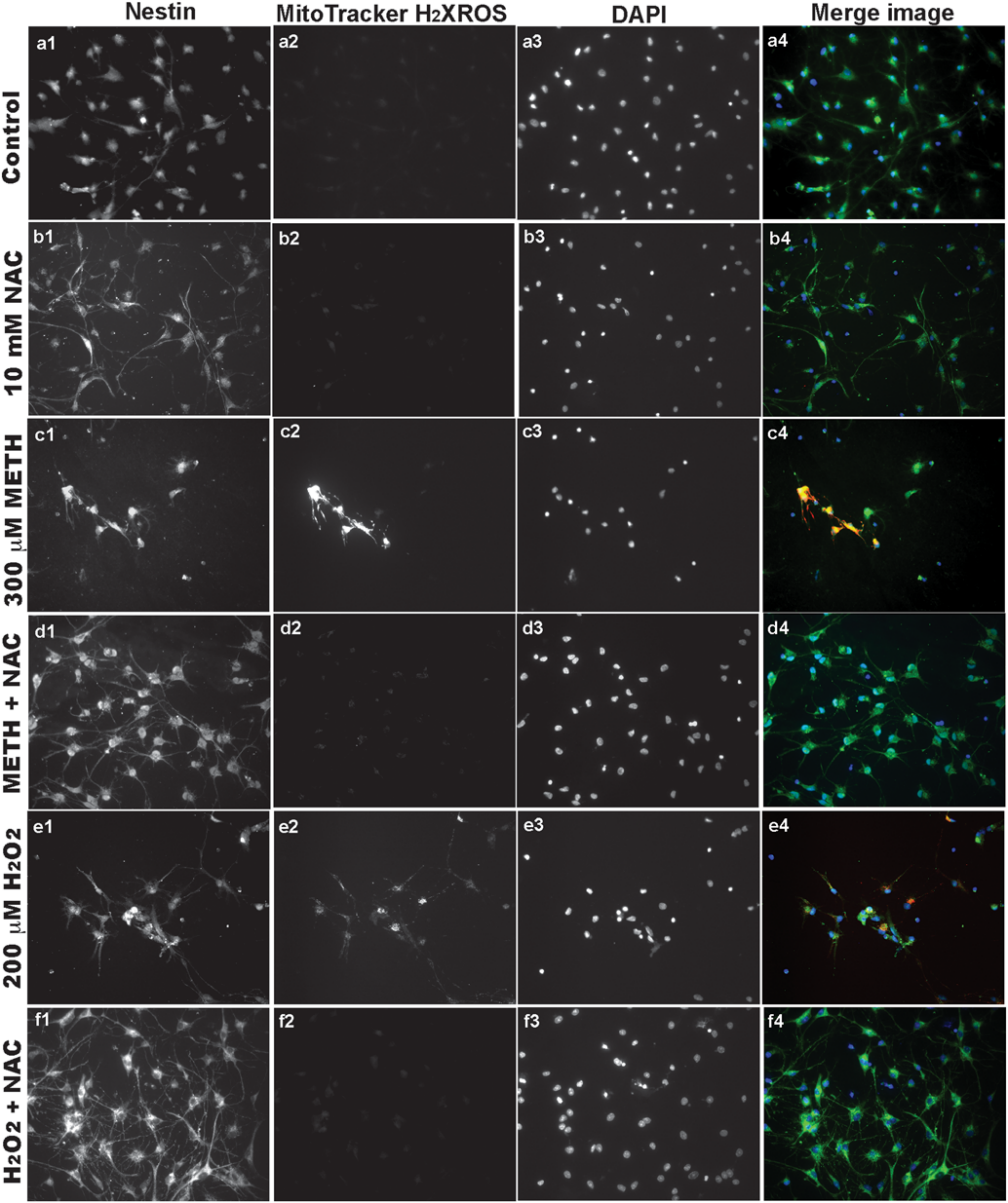
METH increases the generation of ROS in rhNPC. Neurospheres derived from rhNPC were digested and cells were plated on poly-D-lysine-coated cover slips. Cells were treated with 300 µM METH for 24 hours or pre-treated with 10 mM NAC for 30 min, then treated with 300 µM METH for 24 hours. Cells were then subjected to mitochondrial ROS staining with MitoTracker® CM-H_2_ROS (a reduced, nonfluorescent dye that fluoresces upon oxidation) (a2-d2, red), immunostaining with mouse nestin monoclonal antibody (a1-d1, green) and nucleus staining with DAPI (a3-d3, blue) and visualized by Zeiss Axiovert microscope. Cells were treated with 200 µM H_2_O_2_ for 30 min as a positive control (e1-f4).

Next, we examined the ROS production in rhNPC that were pre-treated with 10 mM NAC, and then treated with 300 µM METH or treated with 300 µM METH alone for 24 hours. Cells were subjected to staining with MitoTracker®Red CM-H2XROS which fluoresces upon oxidation and is indicative of ROS generation. We observed that cells with or without NAC treatment had no significant ROS generation (Fig. 7, a2, b2), whereas, ROS were induced by METH treatment (Fig. 7, c2), and this was blocked by pre-treatment with NAC (Fig. 7 d2). H_2_O_2_ treatment mimicked the METH action in an NAC-sensitive manner (Fig. 7, e2, f2). The results suggest that METH can induce ROS generation, but has no effects on Ca^2+^ influx in rhNPC.

### METH-mediated mitochondrial fragmentation is associated with the oligomerization and translocation of Drp1 from the cytoplasm to the mitochondria

**Figure 8:**
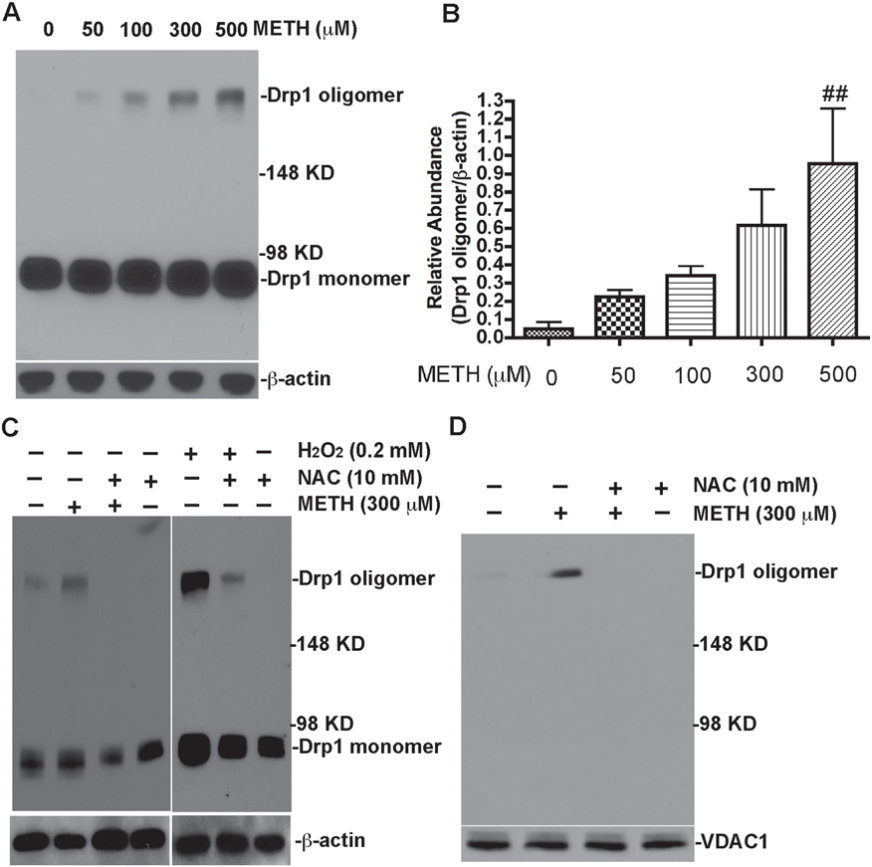
Oxidative stress-mediated oligomerization and translocation of Drp1 contribute to mitochondrial fragmentation. (A) rhNPC were treated with different concentrations of METH as indicated, then subjected to non-reducing SDS-PAGE and western blot assay with Drp1 monoclonal antibody and β-actin as a loading control. The data were quantified by ImageJ software (B) ## denotes *p*<0.05 as compared to control. (C) Cells were treated with 300 µM METH or 200 µM H_2_O_2_ for 24 hours or were pre-incubated with 10 mM NAC for 30 min then with 300 µM METH or 200 µM H_2_O_2_ treatment for 24 hours. They were then subjected to non-reducing SDS-PAGE and western blot assay with Drp1 monoclonal antibody and β-actin as a loading control. (D) Cells were treated with 300 µM METH alone or pre-incubated with 10 mM NAC for 30 min, then with 300 µM METH treatment for 24 hours, and subsequently subjected to cell fractionation and western blotting with Drp1 monoclonal antibody as described in materials and methods. VDAC1 was used as a mitochondrial marker and loading control.

Drp1 has been proposed to encircle mitochondria and function as a drawstring to mediate constriction and scission resulting in the fission of mitochondria [46]. Purified Drp1 has the ability to self-assemble into ring-like multimers on the mitochondrial outer membrane *in vitro* [28], [29], [47]. To explore whether METH-mediated mitochondrial fragmentation is through Drp1 oligomerization induced by oxidative stress, we treated rhNPC with different concentrations of METH for 24 hours and then analyzed the cytosolic Drp1 by non-reducing SDS-PAGE. Our results demonstrated that Drp1 oligomerization was induced by METH treatment in a dose-dependent manner (Fig. 8A; Fig. 8B). Importantly, the Drp1 oligomerization was reversed by NAC pre-incubation (Fig. 8C, left panel) similar to NAC effects on H_2_O_2_ treatment (Fig. 8C, right panel). This is consistent with the METH-induced ROS generation in Fig. 7 and strengthens the association between Drp1 oligomerization and mitochondrial damage.

To further investigate whether METH-mediated mitochondrial fragmentation is caused by increased recruitment of oligomeric Drp1 from the cytoplasm to the mitochondrial outer membrane, we performed *in vitro* subcellular fractionations on untreated or METH-treated rhNPC. Our results showed that METH induced an increase of oligomeric Drp1 in the mitochondrial fraction, whereas NAC prevented the association of METH-induced oligomeric Drp1 with mitochondria (Fig. 8D). These data suggest that METH-mediated mitochondrial fragmentation is not only associated with the formation of Drp1 oligomers but is also determined by the translocation of Drp1 oligomers from the cytosol to the mitochondrial outer membrane. Furthermore, our data demonstrate that METH-induced ROS may be an important mediator for mitochondrial fragmentation.

## Discussion

In this study we observed that METH induced a decrease in proliferation, an increase in apoptosis and a disruption of mitochondrial networks in rhNPC, resulting in mitochondrial fragmentation. Further investigation also suggested that Drp1 was involved in regulating mitochondrial morphology, and that the oligomerization and subsequent translocation of Drp1 contributed to the regulation of METH-mediated mitochondrial fragmentation in which ROS acted as an important mediator.

It has been suggested that the subgranular zone (SGZ) of the dentate gyrus in the hippocampus of the rodent brain is an important source of dividing progenitor cells, and newborn neurons generated from these progenitor cells migrate into the granule cell layer, differentiate, extend axons and express neuronal marker proteins [18]–[21]. The hippocampus is an area of particular interest as it is central to many aspects of the addictive process, including drug use relapse. Moreover, the hippocampus is particularly vulnerable to METH [48], [49]. METH induces apoptosis of striatal glutamic acid decarboxylase-containing neurons due to the interactions between ER stress and mitochondrial death pathways [7]–[11]. In addition to neuronal death, astroglial activation was also found in METH-induced toxicity [12], [13]. Decreased neurogenesis in the SGZ of the hippocampus is another product of opiate and psychostimulant drugs [50]. Neurogenesis in the dentate gyrus is also decreased after repeated administration of psychostimulants such as phencyclidine (PCP), dizocilpine (MK-801), and METH [51]. This inhibition of neurogenesis affects the replacement and repair of damaged cells and may finally contribute to neurodegenerative disease phenotypes. Thus, is necessary to have a better understanding of the contribution of METH abuse to neurodegenerative disorders via its effects on the regeneration of the brain. We utilized rhNPC as a model to examine the effects of METH on cell proliferation. Our results demonstrate that METH inhibits rhNPC proliferation (Fig. 2), which may contribute to decreased neurogenesis. It is well known that cell death of NPCs, alteration of the proliferative environment of the subventricular zone (SVG) and direct action of drugs of abuse on progenitor cells contribute to decreased neurogenesis [50], [52]. Similarly, when treating rhNPC with METH, we found that the frequency of apoptosis was increased (Fig. 3). These results suggest that METH-induced inhibition of rhNPC proliferation is associated with METH-induced apoptosis. Furthermore, the inhibition of proliferation precedes the METH-induced apoptosis. Z-VAD, a pan-caspase inhibitor, has no significant effects on METH-induced inhibition of proliferation but prevents the METH-induced apoptosis (data not shown). These data suggest that the apoptotic cascade may be triggered after rhNPC proliferation is inhibited. Interestingly, we also observed that METH-induced apoptosis was accompanied by the changes in mitochondrial morphology.

Mitochondria are dynamic organelles, frequently dividing and fusing with one another, and the mitochondrial fission and fusion processes participate in apoptosis [46], [53]–[55]. Mitochondrial fission is normally recognized as an early event during apoptosis. Moreover, excessive mitochondrial fragmentation appears to be a prerequisite step in intrinsic apoptosis pathways, as several components of the mitochondrial fission machinery, including Drp1, Fis1, and Endophilin B1, have been implicated in programmed cell death progression [56]. Our results demonstrate that METH induces mitochondrial fragmentation during the early stages of apoptosis (Fig. 3; Fig. 4), which is consistent with reports that this process of fragmentation occurs early in the cell-death pathway [39], [57], [58]. Moreover, the fragmentation of mitochondrial networks induced by METH was found to be mediated by Drp1, a dynamin-related protein which regulates apoptosis-related fission, and inhibition of Drp1 activity by Drp1 siRNA also blocked METH-induced mitochondrial fragmentation (Fig. 5). These results suggest that mitochondrial fragmentation is involved in METH-induced apoptosis and Drp1 plays a crucial role in regulating METH-induced mitochondrial fragmentation in rhNPC.

It is well known that METH produces dopaminergic deficits, in part, by affecting neuronal dopamine transport and inducing reactive species, such as ROS and/or RNS in dopaminergic neurons [59], [60]. Moreover, METH increases Glu levels in the mammalian brain, which can activate ionotropic receptors, such as NMDA and AMPA receptors. This increase in intracellular Ca^2+^ levels and RNS formation [4]–[6] contributes to METH-mediated neurotoxicity. However, our results suggest that METH has no effects on Ca^2+^ influx or AMPA receptor-mediated Ca^2+^ influx induced by glutamate in rhNPC, whereas it does produce an increase in ROS generation in rhNPC (Fig. 6; Fig. 7). These results indicate that oxidative stress elicited by METH plays an important role in METH-induced mitochondrial fragmentation in rhNPC, although some other factors may be involved in this process.

Mitochondria serve as a main source of ROS [61] and also as the target of ROS. Endogenous formation of ROS and alteration of the cells' redox status have been suggested to facilitate cell death [62], [63]. These ROS may target some proteins which are associated with mitochondrial fragmentation and change their structure to direct mitochondrial networks towards fragmentation. Recent studies have suggested that mitochondrial fragmentation may be regulated by a variety of cellular events, including cell division, metabolic flux, cell death and differentiation [64]. Most of these processes seem to alter the post-translational modification of Drp1, including its phosphorylation [65], ubiquitinylation [66] and sumoylation [67], [68]. In this study, we found that the oligomerization and localization of Drp1 in cells was also associated with METH-induced mitochondrial fragmentation (Fig. 8). These findings suggest that METH-induced mitochondrial fragmentation is mediated by ROS, which indirectly regulates the oligomerization and translocation of Drp1 from the cytosol to the mitochondria.

A common theme throughout the diverse dynamin superfamily is that self-assembly and oligomerization play important roles in the function of these proteins [69]–[71]. Although Drp1 exists primarily as a cytosolic homo-tetramer, it can also self-assemble into higher order structures on the mitochondrial outer membrane, where it is required for proper mitochondrial division. How Drp1 forms oligomers and subsequently mediates mitochondrial fragmentation remains to be elucidated. It has been reported that C-terminal GTPase effector (GED) domains are important for stimulation of GTPase activity, formation and stability of higher order complexes and efficient mitochondrial division [47]. We screened the primary structure of Drp1 and found that it contains several cysteine residues that may be sensitive to ROS. The thiol crosslinking agent diamide also induces Drp1 oligomerization [72]. These findings support the idea that oxidative stress plays an important role in the process of Drp1 oligomerization.

Drp1 oligomerization was a prerequisite for mitochondrial fragmentation, and these oligomers cannot function until they translocate from the cytosol to mitochondrial outer membrane. Our results demonstrate that oxidative stress increases the translocation of oligomeric Drp1 to the mitochondrial membrane, and this was reversed by the reducing agent, NAC (Fig. 8). Taken together, our results indicate METH-induced oxidative stress plays an important role in mitochondrial fragmentation, particularly in the process of Drp1 oligomerization and translocation from the cytosol to the mitochondria. These findings will provide insights into understanding the pathogenesis of neurodegenerative disorders related to METH abuse.

## Materials and Methods

### Isolation and Culture of rhNPCs

rhNPC cultures were prepared as reported previously [73], with some modifications. Briefly, brain hippocampi were dissociated from embryonic day 17 (E17) Sprague-Dawley rat embryos following protocols approved previously by the University of Nebraska Medical Center Institutional Animal Care and Use Committee and utilizing National Institutes of Health (NIH) ethical guidelines. After removal of the meninges, the tissue was dissociated mechanically into single cells in a Ca^2+^/Mg^2+^-free Hank's balanced salt solution (HBSS) and cultured at a density of 5×10^6^ cells/flask in 10 ml neuronal progenitor basal medium (NPBM; Lonza BioWalkersville, MD, USA) supplemented with 20 ng/ml basic fibroblast growth factor (bFGF ; BioWalkersville), 20 ng/ml Epidermal growth factor (EGF; BioWalkersville) and 2% neural survival factor-1 (NSF-1; BioWalkersville) in T75 flasks. Neurospheres formed after 4–7 days in culture and were dissociated with Trypsin-EDTA solution (Sigma, St.Louis, MO) for 20 min at 37°C. After washing with HBSS twice, cells were titrated to a single-cell suspension and cultured on poly-D-lysine pre-coated 6-well plates, 24-well plates or plates containing pre-coated cover slips. They were then used for immunostaining assay, Ca^2+^ flux analysis, western blotting, cell cycle analysis and ROS detection.

### Cell cycle assay

Cell cycle was examined by propidium iodide (PI, Sigma) staining [74]. 5×10^6^ cells were harvested and washed twice with Ca^2+^/Mg^2+^ free phosphate-buffered saline (PBS), fixed overnight in 70% cold ethanol, digested with RNase A (Sigma) and stained with PI (100 µg/ml). Data were obtained and analyzed by flow cytometry with the CellQuest software on a FACScan (Becton-Dickinson, New Jersey, USA) using a cell population from which debris were gated out.

### Mitochondrial staining

Cells grown on cover slips were stained with MitoTracker®Red CM-XRos (Invitrogen, Carlsbad, CA) at 37°C in a humidified 5% CO_2_ atmosphere for 15 min and fixed with 3.7% formaldehyde in culture medium for another 15 min at 37°C. After washing with PBS twice, cells were subjected to immunostaining and/or mounted with SlowFade light antifade reagent (Molecular Probes) and analyzed by Zeiss Axiovert microscope.

### Immunofluorescence

Cells grown on cover slips were fixed with 3.7% formaldehyde in culture medium for 15 min at 37°C. After washing with PBS twice, cells were permeabilized with 0.2% Triton X-100 in PBS at 4°C for 10 min, then blocked with 2% bovine serum albumin (BSA) at room temperature for 2 hours. Cells were further incubated for 2 hours at room temperature with antibodies to nestin (1∶200, Chemicon, Temecula, CA), glial fibrillary acidic protein (GFAP, 1∶1,000, polyclonal; DAKO, Carpinteria, CA), β-tubulin Isotype III (1∶400, Sigma) or Drp1 (1∶1000, BD Transduction Laboratories, Lexington, KY), followed by staining with Alexa Fluor® 488 goat anti-mouse IgG or Alexa Fluor® 594 goat anti-rabbit IgG (1∶200; Molecular Probes, Eugene, OR) for 2 h at RT. DAPI (1∶10,000; Sigma) was used (10 min at RT) for nuclear staining. After washing, cells were mounted with SlowFade light antifade reagent (Molecular Probes) and analyzed by Zeiss Axiovert microscope.

### Intracellular calcium measurements

Cells cultured on glass cover slips were loaded with 7.1 µM fura II-AM for 30 min at 37°C in Ringer's solution [145 mM NaCl, 5 mM KCl, 1 mM MgCl_2_, 10 mM HEPES, 2 mM CaCl_2_ and 10 mM D-glucose, pH 7.4]. Cells were then washed twice and incubated again for 20 min in Ringer's solution to allow for intracellular dye cleavage. The cover slips were inserted into the chamber of a PTI Deltascan System, and fura II was excited at wavelengths of 340 and 380 nm as previously described [75], [76].

### ROS production determination

rhNPC grown on cover slips were stained with the reduced mitochondrial dye, MitoTracker®Red CM-H2XROS (Invitrogen), which fluoresces upon oxidation, and were cultured at 37°C in humidified 5% CO_2_ atmosphere for 15 min, and fixed with 3.7% formaldehyde in culture medium for another 15 min at 37°C. After washing with PBS twice, cells were subjected to immunostaining and/or mounted with SlowFade light antifade reagent (Molecular Probes) and analyzed by Zeiss Axiovert microscope.

### siRNA knockdown of Drp1

siRNA knockdown was performed as previously described [77]. Briefly, smart-pool pre-designed siRNA duplexes targeted against rat Drp1 mRNA were synthesized by Dharmacon (Lafayette, CO). rhNPC were plated at a density of 0.5×10^6^ cells/well in 24-well plates (BD Biosciences, San Diego, CA). Cells were transfected with 100 nM siRNA duplex for 24 h in the presence of siIMPORTER (Upstate Cell Signaling Solutions, Charlottesville, VA) according to the manufacturer's instructions. A non-specific control siRNA (Dharmacon, Lafayette, CO) was also transfected at the same concentration as control.

### Non-reducing SDS–PAGE and Western Blot assay

Immunoblotting was performed as described [78]. Briefly, the cells were resuspended in lysis buffer [10 mM HEPES (pH 7.4), 2 mM EGTA, 0.5% NP-40, 1 mM NaF, 1 mM NaVO_4_, 1 mM PMSF, 50 mg/ml trypsin inhibitor, 10 mg/ml aprotinin and leupeptin] and placed on ice for 30 min. The lysates were centrifuged at 12,000×g for 12 min at 4°C, and the protein concentration was determined with BSA as a standard. Equivalent samples (30 µg protein) were subjected to non-reducing SDS-PAGE (without β-mercaptoethanol or DTT in loading buffer) on 6% acrylamide gel. The proteins were then transferred onto nitrocellulose membranes, and probed with primary antibodies (mouse monoclonal anti-DLP1/Drp1, BD Transduction Laboratories, Lexington, KY; β-actin, Sigma-Aldrich, St. Louis, MO, as a loading control) followed by the appropriate secondary antibodies conjugated to horseradish peroxidase (KPL, Gaithersburg, MD, USA). Immunoreactive bands were visualized using enhanced chemiluminescence (Pierce, Rockford, IL). The molecular sizes of the proteins were determined by comparison with pre-stained protein markers (Invitrogen).

### Subcellular fractionation

Cells were fractionated by differential centrifugation as previously described [79]–[81]. Briefly, cells were harvested through Trypsin-EDTA solution digestion, and then centrifuged and resuspended in three volumes of hypotonic buffer [210 mM sucrose, 70 mM mannitol, 10 mM HEPES (pH 7.4), 1 mM EDTA] containing 1 mM phenylmethylsulfonyl fluoride (PMSF), 50 mg/ml trypsin inhibitor, 10 mg/ml leupeptin, 5 mg/ml aprotinin and 10 mg/ml pepstatin. After gentle homogenization with a Dounce homogenizer, cell lysates were centrifuged at 1000×g for 5 min to remove unbroken cells and nuclei, and then cytosolic fractions and the mitochondrial pellets were obtained by further centrifugation at 10,000×g for 30 min.

### Statistical analysis

Data were analyzed as means±SD. The data were evaluated statistically by the analysis of variance (ANOVA). Significance was determined as *p*<0.05 and *p*<0.01.

## References

[1] Hanson GR, Rau KS, Fleckenstein AE (2004). The methamphetamine experience: a NIDA partnership.. Neuropharmacology.

[2] Talloczy Z, Martinez J, Joset D, Ray Y, Gacser A (2008). Methamphetamine inhibits antigen processing, presentation, and phagocytosis.. PLoS Pathog.

[3] Meredith CW, Jaffe C, Ang-Lee K, Saxon AJ (2005). Implications of chronic methamphetamine use: a literature review.. Harv Rev Psychiatry.

[4] Hendrickson H, Laurenzana E, Owens SM (2006). Quantitative determination of total methamphetamine and active metabolites in rat tissue by liquid chromatography with tandem mass spectrometric detection.. Aaps J.

[5] Cadet JL, Krasnova IN, Jayanthi S, Lyles J (2007). Neurotoxicity of substituted amphetamines: molecular and cellular mechanisms.. Neurotox Res.

[6] Simoes PF, Silva AP, Pereira FC, Marques E, Grade S (2007). Methamphetamine induces alterations on hippocampal NMDA and AMPA receptor subunit levels and impairs spatial working memory.. Neuroscience.

[7] Jayanthi S, Deng X, Bordelon M, McCoy MT, Cadet JL (2001). Methamphetamine causes differential regulation of pro-death and anti-death Bcl-2 genes in the mouse neocortex.. Faseb J.

[8] Deng X, Cadet JL (2000). Methamphetamine-induced apoptosis is attenuated in the striata of copper-zinc superoxide dismutase transgenic mice.. Brain Res Mol Brain Res.

[9] Deng X, Jayanthi S, Ladenheim B, Krasnova IN, Cadet JL (2002). Mice with partial deficiency of c-Jun show attenuation of methamphetamine-induced neuronal apoptosis.. Mol Pharmacol.

[10] Deng X, Wang Y, Chou J, Cadet JL (2001). Methamphetamine causes widespread apoptosis in the mouse brain: evidence from using an improved TUNEL histochemical method.. Brain Res Mol Brain Res.

[11] Jayanthi S, Deng X, Noailles PA, Ladenheim B, Cadet JL (2004). Methamphetamine induces neuronal apoptosis via cross-talks between endoplasmic reticulum and mitochondria-dependent death cascades.. Faseb J.

[12] Pubill D, Canudas AM, Pallas M, Camins A, Camarasa J (2003). Different glial response to methamphetamine- and methylenedioxymethamphetamine-induced neurotoxicity.. Naunyn Schmiedebergs Arch Pharmacol.

[13] Miyatake M, Narita M, Shibasaki M, Nakamura A, Suzuki T (2005). Glutamatergic neurotransmission and protein kinase C play a role in neuron-glia communication during the development of methamphetamine-induced psychological dependence.. Eur J Neurosci.

[14] Eriksson PS, Perfilieva E, Bjork-Eriksson T, Alborn AM, Nordborg C (1998). Neurogenesis in the adult human hippocampus.. Nat Med.

[15] Fontana X, Nacher J, Soriano E, del Rio JA (2006). Cell proliferation in the adult hippocampal formation of rodents and its modulation by entorhinal and fimbria-fornix afferents.. Cereb Cortex.

[16] Yoshimura S, Takagi Y, Harada J, Teramoto T, Thomas SS (2001). FGF-2 regulation of neurogenesis in adult hippocampus after brain injury.. Proc Natl Acad Sci U S A.

[17] Kuhn HG, Dickinson-Anson H, Gage FH (1996). Neurogenesis in the dentate gyrus of the adult rat: age-related decrease of neuronal progenitor proliferation.. J Neurosci.

[18] Kaplan MS, Bell DH (1984). Mitotic neuroblasts in the 9-day-old and 11-month-old rodent hippocampus.. J Neurosci.

[19] Kaplan MS, Hinds JW (1977). Neurogenesis in the adult rat: electron microscopic analysis of light radioautographs.. Science.

[20] Stanfield BB, Trice JE (1988). Evidence that granule cells generated in the dentate gyrus of adult rats extend axonal projections.. Exp Brain Res.

[21] Cameron HA, Woolley CS, McEwen BS, Gould E (1993). Differentiation of newly born neurons and glia in the dentate gyrus of the adult rat.. Neuroscience.

[22] Dawirs RR, Teuchert-Noodt G (2001). A novel pharmacological concept in an animal model of psychosis.. Acta Psychiatr Scand.

[23] Abrous DN, Koehl M, Le Moal M (2005). Adult neurogenesis: from precursors to network and physiology.. Physiol Rev.

[24] Huang P, Yu T, Yoon Y (2007). Mitochondrial clustering induced by overexpression of the mitochondrial fusion protein Mfn2 causes mitochondrial dysfunction and cell death.. Eur J Cell Biol.

[25] McBride HM, Neuspiel M, Wasiak S (2006). Mitochondria: more than just a powerhouse.. Curr Biol.

[26] Chan DC (2006). Mitochondrial fusion and fission in mammals.. Annu Rev Cell Dev Biol.

[27] Okamoto K, Shaw JM (2005). Mitochondrial morphology and dynamics in yeast and multicellular eukaryotes.. Annu Rev Genet.

[28] Yoon Y, Pitts KR, McNiven MA (2001). Mammalian dynamin-like protein DLP1 tubulates membranes.. Mol Biol Cell.

[29] Smirnova E, Griparic L, Shurland DL, van der Bliek AM (2001). Dynamin-related protein Drp1 is required for mitochondrial division in mammalian cells.. Mol Biol Cell.

[30] Yu T, Fox RJ, Burwell LS, Yoon Y (2005). Regulation of mitochondrial fission and apoptosis by the mitochondrial outer membrane protein hFis1.. J Cell Sci.

[31] Yoon Y, Krueger EW, Oswald BJ, McNiven MA (2003). The mitochondrial protein hFis1 regulates mitochondrial fission in mammalian cells through an interaction with the dynamin-like protein DLP1.. Mol Cell Biol.

[32] Karbowski M, Jeong SY, Youle RJ (2004). Endophilin B1 is required for the maintenance of mitochondrial morphology.. J Cell Biol.

[33] Waterham HR, Koster J, van Roermund CW, Mooyer PA, Wanders RJ (2007). A lethal defect of mitochondrial and peroxisomal fission.. N Engl J Med.

[34] Zuchner S, Mersiyanova IV, Muglia M, Bissar-Tadmouri N, Rochelle J (2004). Mutations in the mitochondrial GTPase mitofusin 2 cause Charcot-Marie-Tooth neuropathy type 2A.. Nat Genet.

[35] Alexander C, Votruba M, Pesch UE, Thiselton DL, Mayer S (2000). OPA1, encoding a dynamin-related GTPase, is mutated in autosomal dominant optic atrophy linked to chromosome 3q28.. Nat Genet.

[36] Parra V, Eisner V, Chiong M, Criollo A, Moraga F (2007). Changes in mitochondrial dynamics during ceramide-induced cardiomyocyte early apoptosis.. Cardiovasc Res.

[37] Hom JR, Gewandter JS, Michael L, Sheu SS, Yoon Y (2007). Thapsigargin induces biphasic fragmentation of mitochondria through calcium-mediated mitochondrial fission and apoptosis.. J Cell Physiol.

[38] Copp J, Wiley S, Ward MW, van der Geer P (2005). Hypertonic shock inhibits growth factor receptor signaling, induces caspase-3 activation, and causes reversible fragmentation of the mitochondrial network.. Am J Physiol Cell Physiol.

[39] Frank S, Gaume B, Bergmann-Leitner ES, Leitner WW, Robert EG (2001). The role of dynamin-related protein 1, a mediator of mitochondrial fission, in apoptosis.. Dev Cell.

[40] Sugioka R, Shimizu S, Tsujimoto Y (2004). Fzo1, a protein involved in mitochondrial fusion, inhibits apoptosis.. J Biol Chem.

[41] Smirnova E, Shurland DL, Ryazantsev SN, van der Bliek AM (1998). A human dynamin-related protein controls the distribution of mitochondria.. J Cell Biol.

[42] James DI, Parone PA, Mattenberger Y, Martinou JC (2003). hFis1, a novel component of the mammalian mitochondrial fission machinery.. J Biol Chem.

[43] Lee YJ, Jeong SY, Karbowski M, Smith CL, Youle RJ (2004). Roles of the mammalian mitochondrial fission and fusion mediators Fis1, Drp1, and Opa1 in apoptosis.. Mol Biol Cell.

[44] Parone PA, James DI, Da Cruz S, Mattenberger Y, Donze O (2006). Inhibiting the mitochondrial fission machinery does not prevent Bax/Bak-dependent apoptosis.. Mol Cell Biol.

[45] Whitney NP, Peng H, Erdmann NB, Tian C, Monaghan DT (2008). Calcium-permeable AMPA receptors containing Q/R-unedited GluR2 direct human neural progenitor cell differentiation to neurons.. Faseb J.

[46] Youle RJ, Karbowski M (2005). Mitochondrial fission in apoptosis.. Nat Rev Mol Cell Biol.

[47] Zhu PP, Patterson A, Stadler J, Seeburg DP, Sheng M (2004). Intra- and intermolecular domain interactions of the C-terminal GTPase effector domain of the multimeric dynamin-like GTPase Drp1.. J Biol Chem.

[48] Shimazu K, Zhao M, Sakata K, Akbarian S, Bates B (2006). NT-3 facilitates hippocampal plasticity and learning and memory by regulating neurogenesis.. Learn Mem.

[49] Raudensky J, Yamamoto BK (2007). Effects of chronic unpredictable stress and methamphetamine on hippocampal glutamate function.. Brain Res.

[50] Eisch AJ, Harburg GC (2006). Opiates, psychostimulants, and adult hippocampal neurogenesis: Insights for addiction and stem cell biology.. Hippocampus.

[51] Maeda K, Sugino H, Hirose T, Kitagawa H, Nagai T (2007). Clozapine prevents a decrease in neurogenesis in mice repeatedly treated with phencyclidine.. J Pharmacol Sci.

[52] Lee CT, Chen J, Hayashi T, Tsai SY, Sanchez JF (2008). A mechanism for the inhibition of neural progenitor cell proliferation by cocaine.. PLoS Med.

[53] Bereiter-Hahn J, Voth M (1994). Dynamics of mitochondria in living cells: shape changes, dislocations, fusion, and fission of mitochondria.. Microsc Res Tech.

[54] Hermann GJ, Shaw JM (1998). Mitochondrial dynamics in yeast.. Annu Rev Cell Dev Biol.

[55] Chan DC (2006). Mitochondria: dynamic organelles in disease, aging, and development.. Cell.

[56] Suen DF, Norris KL, Youle RJ (2008). Mitochondrial dynamics and apoptosis.. Genes Dev.

[57] Capano M, Crompton M (2002). Biphasic translocation of Bax to mitochondria.. Biochem J.

[58] Karbowski M, Arnoult D, Chen H, Chan DC, Smith CL (2004). Quantitation of mitochondrial dynamics by photolabeling of individual organelles shows that mitochondrial fusion is blocked during the Bax activation phase of apoptosis.. J Cell Biol.

[59] Riddle EL, Fleckenstein AE, Hanson GR (2006). Mechanisms of methamphetamine-induced dopaminergic neurotoxicity.. Aaps J.

[60] Volz TJ, Hanson GR, Fleckenstein AE (2007). The role of the plasmalemmal dopamine and vesicular monoamine transporters in methamphetamine-induced dopaminergic deficits.. J Neurochem.

[61] Turrens JF (2003). Mitochondrial formation of reactive oxygen species.. J Physiol.

[62] Shen HM, Liu ZG (2006). JNK signaling pathway is a key modulator in cell death mediated by reactive oxygen and nitrogen species.. Free Radic Biol Med.

[63] Kowaltowski AJ, Castilho RF, Vercesi AE (2001). Mitochondrial permeability transition and oxidative stress.. FEBS Lett.

[64] Cerveny KL, Tamura Y, Zhang Z, Jensen RE, Sesaki H (2007). Regulation of mitochondrial fusion and division.. Trends Cell Biol.

[65] Cribbs JT, Strack S (2007). Reversible phosphorylation of Drp1 by cyclic AMP-dependent protein kinase and calcineurin regulates mitochondrial fission and cell death.. EMBO Rep.

[66] Karbowski M, Neutzner A, Youle RJ (2007). The mitochondrial E3 ubiquitin ligase MARCH5 is required for Drp1 dependent mitochondrial division.. J Cell Biol.

[67] Harder Z, Zunino R, McBride H (2004). Sumo1 conjugates mitochondrial substrates and participates in mitochondrial fission.. Curr Biol.

[68] Wasiak S, Zunino R, McBride HM (2007). Bax/Bak promote sumoylation of DRP1 and its stable association with mitochondria during apoptotic cell death.. J Cell Biol.

[69] Haller O, Kochs G (2002). Interferon-induced mx proteins: dynamin-like GTPases with antiviral activity.. Traffic.

[70] Danino D, Hinshaw JE (2001). Dynamin family of mechanoenzymes.. Curr Opin Cell Biol.

[71] Sever S (2002). Dynamin and endocytosis.. Curr Opin Cell Biol.

[72] Costantini P, Belzacq AS, Vieira HL, Larochette N, de Pablo MA (2000). Oxidation of a critical thiol residue of the adenine nucleotide translocator enforces Bcl-2-independent permeability transition pore opening and apoptosis.. Oncogene.

[73] Peng H, Huang Y, Rose J, Erichsen D, Herek S (2004). Stromal cell-derived factor 1-mediated CXCR4 signaling in rat and human cortical neural progenitor cells.. J Neurosci Res.

[74] Liao XD, Tang AH, Chen Q, Jin HJ, Wu CH (2003). Role of Ca2+ signaling in initiation of stretch-induced apoptosis in neonatal heart cells.. Biochem Biophys Res Commun.

[75] Munsch T, Deitmer JW (1995). Maintenance of Fura-2 fluorescence in glial cells and neurons of the leech central nervous system.. J Neurosci Methods.

[76] Tian C, Erdmann N, Zhao J, Cao Z, Peng H (2008). HIV-infected macrophages mediate neuronal apoptosis through mitochondrial glutaminase.. J Neurochem.

[77] Peng H, Erdmann N, Whitney N, Dou H, Gorantla S (2006). HIV-1-infected and/or immune activated macrophages regulate astrocyte SDF-1 production through IL-1beta.. Glia.

[78] Chen Q, Chai YC, Mazumder S, Jiang C, Macklis RM (2003). The late increase in intracellular free radical oxygen species during apoptosis is associated with cytochrome c release, caspase activation, and mitochondrial dysfunction.. Cell Death Differ.

[79] Lei X, Chen Y, Du G, Yu W, Wang X (2006). Gossypol induces Bax/Bak-independent activation of apoptosis and cytochrome c release via a conformational change in Bcl-2.. Faseb J.

[80] Chen Q, Gong B, Almasan A (2000). Distinct stages of cytochrome c release from mitochondria: evidence for a feedback amplification loop linking caspase activation to mitochondrial dysfunction in genotoxic stress induced apoptosis.. Cell Death Differ.

[81] Chen Q, Turner J, Watson AJ, Dive C (1997). v-Abl protein tyrosine kinase (PTK) mediated suppression of apoptosis is associated with the up-regulation of Bcl-XL.. Oncogene.

